# Supplementary material for: Hot spot prediction in protein-protein interactions by an ensemble system
Source: BMC Syst Biol. 2018 Dec 31;12(Suppl 9):132. doi: 10.1186/s12918-018-0665-8 (PMC6311905; doi:10.1186/s12918-018-0665-8)
Supplement: Supplementary file 2 — All relevant MATLAB code. A simple Matlab implement of our predictor. (ZIP 19,742 kb) [file 12918_2018_665_MOESM2_ESM.zip › Additional file 2/Read ASA/ASA website/dbMPIKT/3BN9D.html]

 NetSurfP - Protein Surface Accessibility and Secondary Structure Predictions

  

|  |  |  |
| --- | --- | --- |
|  |  | NetSurfP - Protein Surface Accessibility and Secondary Structure Predictions  Technical University of Denmark |

  


---

```
# For publication of results, please cite:
# A generic method for assignment of reliability scores applied to solvent accessibility predictions.
# Bent Petersen, Thomas Nordahl Petersen, Pernille Andersen, Morten Nielsen and Claus Lundegaard
# BMC Structural Biology 2009, 9:51 doi:10.1186/1472-6807-9-51
#
# Column 1: Class assignment - B for buried or E for Exposed - Threshold: 25% exposure, but not based on RSA
# Column 2: Amino acid
# Column 3: Sequence name
# Column 4: Amino acid number
# Column 5: Relative Surface Accessibility - RSA
# Column 6: Absolute Surface Accessibility
# Column 7: Z-fit score for RSA prediction
# Column 8: Probability for Alpha-Helix
# Column 9: Probability for Beta-strand
# Column 10: Probability for Coil
E Q  3BN9D                  1    0.823 146.988   1.347   0.003   0.003   0.994
B V  3BN9D                  2    0.202  30.986  -0.165   0.004   0.616   0.381
E Q  3BN9D                  3    0.434  77.512   1.337   0.002   0.816   0.182
B L  3BN9D                  4    0.153  27.941   0.629   0.002   0.816   0.182
E V  3BN9D                  5    0.390  59.866   0.519   0.021   0.756   0.223
B Q  3BN9D                  6    0.244  43.596   0.573   0.004   0.514   0.481
E S  3BN9D                  7    0.420  49.201  -0.495   0.004   0.197   0.799
E G  3BN9D                  8    0.510  40.161  -1.864   0.018   0.088   0.893
E G  3BN9D                  9    0.531  41.813  -2.354   0.018   0.047   0.935
E G  3BN9D                 10    0.532  41.837  -1.712   0.019   0.141   0.840
E L  3BN9D                 11    0.402  73.533  -0.822   0.022   0.359   0.619
E V  3BN9D                 12    0.303  46.633   0.718   0.021   0.451   0.528
E K  3BN9D                 13    0.439  90.302   1.160   0.020   0.205   0.775
E P  3BN9D                 14    0.453  64.238  -0.499   0.018   0.047   0.935
E G  3BN9D                 15    0.596  46.921  -1.950   0.005   0.045   0.951
E G  3BN9D                 16    0.416  32.779  -0.591   0.004   0.138   0.858
E S  3BN9D                 17    0.420  49.271  -0.516   0.004   0.420   0.576
B L  3BN9D                 18    0.155  28.381   0.950   0.002   0.816   0.182
E R  3BN9D                 19    0.408  93.432   0.811   0.001   0.900   0.099
B L  3BN9D                 20    0.080  14.703   0.606   0.001   0.900   0.099
B S  3BN9D                 21    0.167  19.572   0.412   0.001   0.900   0.099
B C  3BN9D                 22    0.046   6.458   0.185   0.001   0.900   0.099
B A  3BN9D                 23    0.306  33.688   0.433   0.001   0.900   0.099
B A  3BN9D                 24    0.099  10.888  -0.146   0.002   0.816   0.182
B S  3BN9D                 25    0.191  22.362  -0.344   0.004   0.514   0.481
B G  3BN9D                 26    0.246  19.321  -1.732   0.004   0.197   0.799
B F  3BN9D                 27    0.248  49.814  -0.838   0.005   0.336   0.660
B T  3BN9D                 28    0.177  24.578  -1.075   0.022   0.552   0.426
B F  3BN9D                 29    0.113  22.739  -0.596   0.021   0.451   0.528
B S  3BN9D                 30    0.213  24.964  -1.061   0.021   0.279   0.699
B S  3BN9D                 31    0.294  34.445  -1.226   0.019   0.141   0.840
B Y  3BN9D                 32    0.114  24.405  -0.477   0.019   0.141   0.840
B A  3BN9D                 33    0.095  10.447  -0.091   0.021   0.279   0.699
B M  3BN9D                 34    0.032   6.423   0.108   0.023   0.655   0.322
B S  3BN9D                 35    0.057   6.704  -0.059   0.023   0.655   0.322
B W  3BN9D                 36    0.040   9.692   0.140   0.021   0.756   0.223
B V  3BN9D                 37    0.041   6.256   0.231   0.021   0.756   0.223
B R  3BN9D                 38    0.121  27.640   0.557   0.023   0.655   0.322
B Q  3BN9D                 39    0.107  19.021   0.473   0.022   0.359   0.619
B A  3BN9D                 40    0.222  24.519   0.645   0.018   0.047   0.935
E P  3BN9D                 41    0.390  55.398   0.448   0.005   0.015   0.979
E G  3BN9D                 42    0.595  46.858  -1.867   0.018   0.019   0.964
E K  3BN9D                 43    0.387  79.565   0.556   0.018   0.019   0.964
E G  3BN9D                 44    0.436  34.321  -0.473   0.018   0.088   0.893
B L  3BN9D                 45    0.160  29.278   0.937   0.005   0.336   0.660
B E  3BN9D                 46    0.086  15.094  -0.324   0.003   0.718   0.279
B W  3BN9D                 47    0.050  12.001  -0.204   0.001   0.900   0.099
B V  3BN9D                 48    0.027   4.104   0.650   0.001   0.959   0.040
B S  3BN9D                 49    0.018   2.145  -0.302   0.001   0.959   0.040
B A  3BN9D                 50    0.076   8.375  -0.746   0.001   0.959   0.040
B I  3BN9D                 51    0.025   4.643   0.665   0.001   0.959   0.040
B S  3BN9D                 52    0.140  16.373   0.597   0.002   0.816   0.182
B G  3BN9D                 53    0.251  19.754  -1.164   0.005   0.336   0.660
E S  3BN9D                 54    0.522  61.120  -1.710   0.005   0.015   0.979
E G  3BN9D                 55    0.574  45.174  -2.678   0.003   0.003   0.994
E G  3BN9D                 56    0.541  42.577  -2.112   0.018   0.019   0.964
E S  3BN9D                 57    0.499  58.459  -0.527   0.018   0.088   0.893
E T  3BN9D                 58    0.296  41.055   0.107   0.020   0.205   0.775
E Y  3BN9D                 59    0.435  93.045  -0.025   0.021   0.279   0.699
B Y  3BN9D                 60    0.189  40.432   0.553   0.020   0.205   0.775
B A  3BN9D                 61    0.251  27.682  -0.264   0.052   0.084   0.864
E D  3BN9D                 62    0.630  90.754  -0.663   0.053   0.043   0.903
E S  3BN9D                 63    0.383  44.841  -0.080   0.052   0.084   0.864
B V  3BN9D                 64    0.093  14.294  -0.096   0.019   0.141   0.840
E K  3BN9D                 65    0.395  81.354   0.838   0.020   0.205   0.775
B G  3BN9D                 66    0.191  15.008  -1.602   0.005   0.336   0.660
B R  3BN9D                 67    0.203  46.601   0.621   0.003   0.718   0.279
B F  3BN9D                 68    0.045   9.132   0.184   0.001   0.900   0.099
B T  3BN9D                 69    0.112  15.507   0.542   0.001   0.900   0.099
B I  3BN9D                 70    0.043   7.863   0.638   0.001   0.900   0.099
B S  3BN9D                 71    0.105  12.294   0.494   0.002   0.816   0.182
E R  3BN9D                 72    0.273  62.471   0.822   0.022   0.552   0.426
B D  3BN9D                 73    0.275  39.700  -0.413   0.022   0.359   0.619
E N  3BN9D                 74    0.444  64.987  -0.035   0.018   0.088   0.893
B S  3BN9D                 75    0.220  25.819  -1.958   0.018   0.088   0.893
E K  3BN9D                 76    0.525 108.034   1.036   0.019   0.141   0.840
B N  3BN9D                 77    0.260  38.049  -0.239   0.022   0.359   0.619
B T  3BN9D                 78    0.182  25.271   1.074   0.018   0.846   0.136
B L  3BN9D                 79    0.073  13.330  -0.827   0.011   0.918   0.071
B Y  3BN9D                 80    0.176  37.526   0.442   0.011   0.918   0.071
B L  3BN9D                 81    0.058  10.656   0.743   0.011   0.918   0.071
B Q  3BN9D                 82    0.177  31.576  -0.334   0.018   0.846   0.136
B M  3BN9D                 83    0.091  18.149  -0.281   0.023   0.655   0.322
E S  3BN9D                 84    0.378  44.348  -0.142   0.022   0.359   0.619
E S  3BN9D                 85    0.385  45.099  -0.796   0.020   0.205   0.775
B L  3BN9D                 86    0.121  22.118   0.077   0.018   0.088   0.893
E R  3BN9D                 87    0.468 107.264   1.030   0.018   0.047   0.935
E A  3BN9D                 88    0.395  43.529  -0.806   0.052   0.084   0.864
E E  3BN9D                 89    0.465  81.288  -0.665   0.053   0.043   0.903
B D  3BN9D                 90    0.163  23.546  -0.853   0.052   0.084   0.864
B T  3BN9D                 91    0.107  14.799   0.243   0.066   0.296   0.638
B A  3BN9D                 92    0.037   4.121  -0.311   0.085   0.775   0.140
B V  3BN9D                 93    0.066  10.083   1.001   0.018   0.846   0.136
B Y  3BN9D                 94    0.038   8.078   0.797   0.018   0.846   0.136
B Y  3BN9D                 95    0.058  12.352   1.051   0.018   0.846   0.136
B C  3BN9D                 96    0.017   2.331   1.533   0.021   0.756   0.223
B A  3BN9D                 97    0.033   3.637  -0.091   0.022   0.552   0.426
B R  3BN9D                 98    0.165  37.877   0.384   0.022   0.359   0.619
E P  3BN9D                 99    0.313  44.415  -0.262   0.020   0.205   0.775
E Y  3BN9D                100    0.356  76.141  -0.656   0.018   0.088   0.893
E L  3BN9D                101    0.353  64.671  -1.336   0.018   0.088   0.893
E T  3BN9D                102    0.473  65.661  -1.672   0.020   0.205   0.775
E Y  3BN9D                103    0.424  90.502  -1.459   0.019   0.141   0.840
E P  3BN9D                104    0.427  60.577  -2.113   0.018   0.088   0.893
E Q  3BN9D                105    0.366  65.350  -1.945   0.018   0.088   0.893
E R  3BN9D                106    0.406  93.043  -2.142   0.018   0.088   0.893
E R  3BN9D                107    0.384  87.936  -2.079   0.018   0.088   0.893
E G  3BN9D                108    0.342  26.884  -2.343   0.018   0.088   0.893
B P  3BN9D                109    0.312  44.202  -2.045   0.018   0.088   0.893
E Q  3BN9D                110    0.349  62.331  -1.684   0.018   0.088   0.893
E N  3BN9D                111    0.407  59.629  -1.384   0.018   0.047   0.935
E V  3BN9D                112    0.363  55.716  -1.351   0.018   0.047   0.935
B S  3BN9D                113    0.293  34.304  -1.048   0.018   0.047   0.935
B P  3BN9D                114    0.145  20.618  -1.103   0.019   0.141   0.840
B F  3BN9D                115    0.058  11.580  -0.164   0.019   0.141   0.840
B D  3BN9D                116    0.089  12.782  -0.494   0.020   0.205   0.775
B N  3BN9D                117    0.063   9.223  -0.156   0.021   0.279   0.699
B W  3BN9D                118    0.048  11.592  -0.451   0.020   0.205   0.775
B G  3BN9D                119    0.039   3.061  -1.881   0.019   0.141   0.840
B Q  3BN9D                120    0.170  30.362   0.037   0.004   0.085   0.910
B G  3BN9D                121    0.097   7.602  -1.322   0.005   0.336   0.660
B T  3BN9D                122    0.073  10.125   0.026   0.004   0.616   0.381
E M  3BN9D                123    0.219  43.742   0.124   0.002   0.816   0.182
B V  3BN9D                124    0.039   5.979   0.377   0.001   0.900   0.099
B T  3BN9D                125    0.093  12.899   0.382   0.001   0.900   0.099
B V  3BN9D                126    0.075  11.558  -0.153   0.002   0.816   0.182
B S  3BN9D                127    0.148  17.392  -0.307   0.004   0.616   0.381
B S  3BN9D                128    0.207  24.284  -0.441   0.004   0.197   0.799
E A  3BN9D                129    0.443  48.819  -1.600   0.018   0.047   0.935
E S  3BN9D                130    0.431  50.490  -0.564   0.018   0.047   0.935
E T  3BN9D                131    0.363  50.362  -0.962   0.018   0.047   0.935
E K  3BN9D                132    0.574 117.990  -1.163   0.018   0.019   0.964
E G  3BN9D                133    0.307  24.145  -1.473   0.018   0.019   0.964
B P  3BN9D                134    0.251  35.631  -0.801   0.018   0.019   0.964
E S  3BN9D                135    0.584  68.433  -1.206   0.018   0.047   0.935
E V  3BN9D                136    0.512  78.664  -2.231   0.018   0.088   0.893
B F  3BN9D                137    0.231  46.362  -0.760   0.018   0.088   0.893
E P  3BN9D                138    0.458  64.948  -1.546   0.019   0.141   0.840
E L  3BN9D                139    0.311  56.853  -1.322   0.020   0.205   0.775
E A  3BN9D                140    0.443  48.830  -1.361   0.020   0.205   0.775
B P  3BN9D                141    0.176  24.932  -2.188   0.004   0.085   0.910
E S  3BN9D                142    0.479  56.139  -1.917   0.004   0.138   0.858
B S  3BN9D                143    0.212  24.835  -2.869   0.004   0.085   0.910
E K  3BN9D                144    0.563 115.727  -2.119   0.005   0.045   0.951
E S  3BN9D                145    0.429  50.220  -1.762   0.005   0.015   0.979
E T  3BN9D                146    0.493  68.407  -1.855   0.005   0.015   0.979
E S  3BN9D                147    0.602  70.508  -2.393   0.003   0.003   0.994
E G  3BN9D                148    0.685  53.894  -2.523   0.003   0.003   0.994
E G  3BN9D                149    0.472  37.162  -1.373   0.005   0.015   0.979
E T  3BN9D                150    0.408  56.659  -0.266   0.005   0.336   0.660
B A  3BN9D                151    0.087   9.631   0.240   0.002   0.816   0.182
B A  3BN9D                152    0.123  13.555   0.288   0.001   0.959   0.040
B L  3BN9D                153    0.034   6.225   0.000   0.001   0.959   0.040
B G  3BN9D                154    0.056   4.376   0.425   0.001   0.959   0.040
B C  3BN9D                155    0.030   4.282   0.376   0.001   0.900   0.099
B L  3BN9D                156    0.088  16.204   0.664   0.001   0.900   0.099
B V  3BN9D                157    0.071  10.867  -1.031   0.004   0.616   0.381
E K  3BN9D                158    0.292  60.044   0.056   0.005   0.336   0.660
E D  3BN9D                159    0.497  71.618  -0.711   0.004   0.085   0.910
B Y  3BN9D                160    0.309  66.097  -1.356   0.005   0.045   0.951
E F  3BN9D                161    0.421  84.495  -1.217   0.005   0.015   0.979
E P  3BN9D                162    0.447  63.486  -0.942   0.005   0.015   0.979
E E  3BN9D                163    0.628 109.694  -0.726   0.005   0.015   0.979
E P  3BN9D                164    0.278  39.491  -1.434   0.004   0.085   0.910
B V  3BN9D                165    0.214  32.938   0.053   0.004   0.616   0.381
B T  3BN9D                166    0.274  37.990  -0.033   0.001   0.900   0.099
B V  3BN9D                167    0.051   7.869   0.572   0.001   0.959   0.040
B S  3BN9D                168    0.193  22.666   0.396   0.001   0.959   0.040
B W  3BN9D                169    0.096  23.016   0.418   0.001   0.900   0.099
E N  3BN9D                170    0.364  53.304   0.380   0.002   0.816   0.182
E S  3BN9D                171    0.450  52.775  -0.665   0.005   0.336   0.660
E G  3BN9D                172    0.594  46.724  -1.199   0.018   0.088   0.893
E A  3BN9D                173    0.583  64.269  -1.053   0.018   0.019   0.964
E L  3BN9D                174    0.459  84.006  -0.214   0.018   0.047   0.935
E T  3BN9D                175    0.527  73.109  -1.595   0.018   0.047   0.935
E S  3BN9D                176    0.509  59.667  -0.416   0.018   0.047   0.935
E G  3BN9D                177    0.383  30.118  -0.986   0.019   0.141   0.840
B V  3BN9D                178    0.117  17.921  -0.906   0.022   0.359   0.619
E H  3BN9D                179    0.437  79.399   0.233   0.023   0.655   0.322
E T  3BN9D                180    0.451  62.581  -0.334   0.022   0.552   0.426
B F  3BN9D                181    0.264  53.025   0.763   0.021   0.279   0.699
B P  3BN9D                182    0.200  28.380  -0.281   0.052   0.084   0.864
E A  3BN9D                183    0.357  39.308  -1.127   0.056   0.142   0.802
E V  3BN9D                184    0.314  48.246  -0.606   0.022   0.359   0.619
B L  3BN9D                185    0.209  38.250  -0.852   0.021   0.451   0.528
E Q  3BN9D                186    0.413  73.708  -0.832   0.022   0.359   0.619
E S  3BN9D                187    0.608  71.293  -3.090   0.019   0.141   0.840
E S  3BN9D                188    0.560  65.597  -1.424   0.018   0.088   0.893
E G  3BN9D                189    0.495  38.988  -2.241   0.018   0.088   0.893
E L  3BN9D                190    0.399  73.130  -0.950   0.020   0.205   0.775
B Y  3BN9D                191    0.297  63.405  -1.285   0.021   0.451   0.528
B S  3BN9D                192    0.160  18.787  -1.215   0.023   0.655   0.322
B L  3BN9D                193    0.185  33.874  -1.032   0.018   0.846   0.136
B S  3BN9D                194    0.077   9.036  -0.889   0.018   0.846   0.136
B S  3BN9D                195    0.062   7.313  -0.358   0.001   0.900   0.099
B V  3BN9D                196    0.090  13.818  -0.310   0.001   0.900   0.099
B V  3BN9D                197    0.155  23.900  -0.490   0.011   0.918   0.071
B T  3BN9D                198    0.185  25.659   0.143   0.021   0.756   0.223
B V  3BN9D                199    0.198  30.356   0.627   0.023   0.655   0.322
E P  3BN9D                200    0.411  58.307  -0.336   0.022   0.359   0.619
E S  3BN9D                201    0.324  37.938  -0.566   0.020   0.205   0.775
E S  3BN9D                202    0.568  66.558  -0.193   0.021   0.279   0.699
E S  3BN9D                203    0.613  71.808  -0.144   0.020   0.205   0.775
E L  3BN9D                204    0.328  60.130  -0.511   0.018   0.088   0.893
E G  3BN9D                205    0.664  52.280  -0.268   0.018   0.047   0.935
E T  3BN9D                206    0.625  86.715   0.413   0.005   0.045   0.951
E Q  3BN9D                207    0.505  90.193   0.496   0.005   0.336   0.660
E T  3BN9D                208    0.511  70.862   1.306   0.002   0.816   0.182
B Y  3BN9D                209    0.061  12.993   0.946   0.001   0.959   0.040
E I  3BN9D                210    0.307  56.703   1.795   0.001   0.959   0.040
B C  3BN9D                211    0.021   2.906   0.533   0.001   0.959   0.040
E N  3BN9D                212    0.317  46.467   0.894   0.001   0.900   0.099
B V  3BN9D                213    0.133  20.396   0.266   0.001   0.900   0.099
E N  3BN9D                214    0.517  75.616   0.159   0.001   0.900   0.099
B H  3BN9D                215    0.243  44.165   0.116   0.004   0.514   0.481
E K  3BN9D                216    0.527 108.424  -0.854   0.004   0.085   0.910
E P  3BN9D                217    0.630  89.454  -2.083   0.005   0.045   0.951
E S  3BN9D                218    0.512  60.030  -1.350   0.004   0.138   0.858
E N  3BN9D                219    0.385  56.408  -1.417   0.005   0.262   0.733
E T  3BN9D                220    0.367  50.889  -0.625   0.004   0.197   0.799
E K  3BN9D                221    0.521 107.252  -1.303   0.005   0.262   0.733
E V  3BN9D                222    0.295  45.311  -1.467   0.005   0.336   0.660
E D  3BN9D                223    0.607  87.425  -1.896   0.004   0.514   0.481
E K  3BN9D                224    0.276  56.711   0.643   0.004   0.616   0.381
E K  3BN9D                225    0.570 117.228   0.605   0.004   0.616   0.381
B V  3BN9D                226    0.151  23.270   0.490   0.023   0.655   0.322
E E  3BN9D                227    0.518  90.477   0.371   0.022   0.552   0.426
B P  3BN9D                228    0.224  31.729  -0.672   0.022   0.359   0.619
E K  3BN9D                229    0.557 114.513  -0.297   0.021   0.279   0.699
E S  3BN9D                230    0.411  48.134  -0.333   0.019   0.141   0.840
E C  3BN9D                231    0.313  44.001  -1.076   0.018   0.088   0.893
E A  3BN9D                232    0.633  69.713  -1.702   0.018   0.088   0.893
E A  3BN9D                233    0.450  49.590  -0.918   0.018   0.088   0.893
E A  3BN9D                234    0.561  61.877  -1.544   0.018   0.047   0.935
E H  3BN9D                235    0.324  58.954  -0.861   0.005   0.045   0.951
E H  3BN9D                236    0.441  80.291  -1.079   0.005   0.045   0.951
B H  3BN9D                237    0.196  35.689  -0.754   0.004   0.085   0.910
B H  3BN9D                238    0.202  36.671  -0.867   0.004   0.085   0.910
B H  3BN9D                239    0.241  43.856  -0.908   0.005   0.045   0.951
B H  3BN9D                240    0.251  45.621  -1.318   0.005   0.015   0.979
B G  3BN9D                241    0.280  22.012  -1.602   0.018   0.019   0.964
B A  3BN9D                242    0.227  25.015  -1.326   0.016   0.005   0.979
E A  3BN9D                243    0.336  37.071  -0.763   0.115   0.016   0.868
E E  3BN9D                244    0.310  54.122  -0.373   0.257   0.016   0.727
E Q  3BN9D                245    0.430  76.834  -0.501   0.339   0.016   0.645
E K  3BN9D                246    0.432  88.945   0.696   0.354   0.048   0.598
B L  3BN9D                247    0.107  19.592  -0.313   0.386   0.097   0.517
B I  3BN9D                248    0.148  27.362   0.264   0.386   0.097   0.517
E S  3BN9D                249    0.289  33.859  -0.227   0.386   0.097   0.517
E E  3BN9D                250    0.349  60.900  -0.872   0.502   0.102   0.396
E E  3BN9D                251    0.353  61.722  -0.513   0.455   0.046   0.498
E D  3BN9D                252    0.368  53.000  -0.864   0.354   0.048   0.598
B L  3BN9D                253    0.198  36.309  -1.235   0.257   0.016   0.727
E N  3BN9D                254    0.445  65.177  -0.570   0.181   0.016   0.803
E G  3BN9D                255    0.524  41.215  -0.884   0.115   0.016   0.868
E A  3BN9D                256    0.484  53.370  -1.796   0.115   0.016   0.868
E A  3BN9D                257    0.800  88.116  -0.737   0.003   0.003   0.994

Explain the output.  Go back.
```

---
